# Therapeutic Plasma Exchange for Severe Exogenous Thyrotoxicosis Following Massive Levothyroxine Ingestion: A Case Report

**DOI:** 10.7759/cureus.105194

**Published:** 2026-03-13

**Authors:** Anas Kartoumah, Adam Abow Alkhier, Laith Kabbani, Noah Baranski, Muaz Mallah, Mohanad Horani, Mohamad Horani

**Affiliations:** 1 Biomedical Sciences, University of South Florida, Tampa, USA; 2 Biomedical Engineering, Arizona State University, Phoenix, USA; 3 Biological Sciences, Arizona State University, Phoenix, USA; 4 Cybersecurity, University of Advancing Technology, Tempe, USA; 5 Internal Medicine, Chandler Regional Medical Center, Chandler, USA

**Keywords:** atrial fibrillation, cholestyramine, dexamethasone, exogenous thyrotoxicosis, levothyroxine overdose, plasmapheresis, tachyarrhythmia, therapeutic plasma exchange, thyroid storm, toxicology

## Abstract

Levothyroxine is among the most commonly prescribed medications worldwide and is generally safe at therapeutic doses; however, massive ingestion can result in severe exogenous thyrotoxicosis and delayed progression to thyroid storm. Management is challenging because clinical deterioration may occur days after ingestion due to the peripheral conversion of thyroxine (T4) to triiodothyronine (T3) and standard medical therapies do not directly remove circulating hormone. We report the case of a 70-year-old male patient with a massive levothyroxine overdose involving the ingestion of approximately 8,000 mcg, leading to worsening thyrotoxicosis with features concerning impending thyroid storm treated with beta-blockade, corticosteroids, and cholestyramine. Given the large intravascular burden of protein-bound T4 and clinical deterioration, therapeutic plasma exchange (TPE) was initiated. Serial TPE resulted in rapid biochemical improvement with marked reductions in free T4 levels and corresponding clinical stabilization. This case highlights the delayed and potentially misleading presentation of massive levothyroxine ingestion and demonstrates the effectiveness of TPE as a rescue therapy in severe exogenous thyrotoxicosis. Early recognition, prolonged monitoring, and consideration of TPE are essential when life-threatening symptoms emerge following massive levothyroxine overdose.

## Introduction

Levothyroxine is among the most commonly prescribed medications worldwide for the treatment of hypothyroidism and is generally safe when taken at therapeutic doses [1]. However, very large ingestions can lead to severe exogenous thyrotoxicosis and, in rare cases, thyroid storm. Thyroid storm is a life-threatening condition caused by extreme excess of thyroid hormones and is marked by high fever, agitation or confusion, rapid heart rate, gastrointestinal symptoms, and heart failure. Thyroid storm remains one of the most dangerous endocrine emergencies, and if not treated quickly, it carries a high risk of death [2].

In exogenous thyrotoxicosis from levothyroxine overdose, clinical deterioration may be delayed because levothyroxine must first be absorbed and then converted peripherally to the more active hormone triiodothyronine (T3). Standard medical therapies, such as corticosteroids and bile acid sequestrants, aim to inhibit or reduce the conversion of hormones, but cannot directly remove already absorbed hormones from circulation [3].

Therapeutic plasma exchange (TPE) has emerged as an adjunctive strategy in severe thyrotoxicosis, especially when conventional treatment is ineffective, contraindicated, or too slow to produce clinical improvement. TPE removes protein-bound thyroid hormones from the bloodstream, leading to rapid reductions in free thyroxine (T4) and free T3 levels [4].

Case reports and smaller clinical series have documented the successful use of TPE in both refractory endogenous thyrotoxicosis and poisoning scenarios involving massive hormone ingestion, including intentional overdose treated with combined hormone and drug toxicities [4,5]. Despite these reports, evidence for TPE in severe exogenous thyrotoxicosis from levothyroxine overdose remains limited, particularly in cases complicated by co-ingestants. This case report contributes to that growing clinical experience by describing successful biochemical and clinical improvement following serial TPE in a patient with impending thyroid storm after massive levothyroxine ingestion.

## Case presentation

A 70-year-old male patient with a history of hypothyroidism, major depressive disorder (recurrent), anxiety, chronic kidney disease, gastroesophageal reflux disease, hiatal hernia, fatty liver, cognitive impairment, dysphagia, dizziness, and peripheral neuropathy presented to the emergency department after an intentional ingestion of approximately 8,000 mcg of levothyroxine (40 200 mcg tablets) and 90 tablets of 10 mg dextroamphetamine-amphetamine (Adderall) in the afternoon on hospital day 1 earlier that day. Per collateral history, he also ingested multiple sedative/hypnotic tablets of unknown specific agents and doses, with urine toxicology later positive for amphetamines, benzodiazepines, tetrahydrocannabinol (THC), and fentanyl.

The massive amphetamine co-ingestion created a diagnostic challenge early in the hospital course. The patient's initial agitation and tachycardia could plausibly reflect sympathomimetic toxicity, but the persistence and progression of cardiovascular instability, including atrial fibrillation with rapid ventricular response (AF with RVR), coupled with rising thyroid hormone levels consistent with severe thyrotoxicosis, shifted concern toward evolving thyroid storm from delayed levothyroxine toxicity.

Initial evaluation and early hospital course

On arrival, the patient was initially clinically stable and underwent evaluation for psychiatric placement. Initial laboratory findings are summarized in Table 1. These demonstrated high anion gap metabolic acidosis, suppressed thyroid-stimulating hormone (TSH) with markedly elevated free T4, and a urine toxicology screen positive for multiple co-ingestants. He received supportive care and electrolyte replacement with improvement in bicarbonate to approximately 14 mmol/L on subsequent testing. He remained afebrile during this period.

**Table 1:** Initial laboratory findings and urine toxicology TSH: thyroid-stimulating hormone; CO₂: carbon dioxide; BUN: blood urea nitrogen; CKD: chronic kidney disease; ALT: alanine transaminase; AST: aspartate aminotransferase; WBC: white blood cell; INR: international normalized ratio; PT: prothrombin time; THC: tetrahydrocannabinol; PCP: phencyclidine; T4: thyroxine

Test	Result	Reference range	Interpretation
Thyroid studies			
TSH	0.171 µIU/mL	0.35-4.94 µIU/mL	Suppressed
Free T4	>4.00 ng/dL	0.70-1.48 ng/dL	Markedly elevated
Basic metabolic panel			
Sodium	142 mmol/L	136-145 mmol/L	Normal
Potassium	3.5 mmol/L	3.5-5.1 mmol/L	Low-normal
Chloride	117 mmol/L	98-107 mmol/L	Elevated
CO₂ (bicarbonate)	11 mmol/L	22-29 mmol/L	Markedly low
Anion gap	18 mEq/L	4-12 mEq/L	Elevated
Glucose	101 mg/dL	70-99 mg/dL	Mildly elevated (stress)
BUN	18 mg/dL	7-20 mg/dL	Normal
Creatinine	1.16 mg/dL	0.7-1.3 mg/dL	Mildly elevated (CKD baseline)
Calcium	8.7 mg/dL	8.6-10.2 mg/dL	Normal
Total bilirubin	1.6 mg/dL	0.2-1.2 mg/dL	Mildly elevated
ALT	19 U/L	7-56 U/L	Normal
AST	13 U/L	10-40 U/L	Normal
Complete blood count			
WBC	8.9×10³/µL	4.0-11.0×10³/µL	Normal
Hemoglobin	9.3 g/dL	13.5-17.5 g/dL	Low (anemia)
Hematocrit	26.4%	41-53%	Low
Platelets	100×10³/µL	150-400×10³/µL	Low (thrombocytopenia)
Neutrophils	90.6%	40-70%	Elevated
Lymphocytes	4.5%	20-40%	Decreased
Coagulation			
INR	1.73	0.8-1.2	Elevated
PT	20.1 sec	11-13.5 sec	Prolonged
Cardiac			
hs-Troponin I	<4 ng/L	<14 ng/L	Normal
Toxicology			
Ethanol	<10 mg/dL	Negative	No significant ethanol
Amphetamines	Positive	Negative	Consistent with reported ingestion
Benzodiazepines	Positive	Negative	Co-ingestion
THC	Positive	Negative	Recent/prior use
Fentanyl	Positive	Negative	Co-ingestion
Barbiturates	Negative	Negative	-
Cocaine	Negative	Negative	-
Methadone	Negative	Negative	-
Opiates	Negative	Negative	-
Oxycodone	Negative	Negative	-
PCP	Negative	Negative	-

During early hospitalization, he developed progressive tachycardia to 140-150 beats/min and AF with RVR, accompanied by agitation and confusion. Thyroid function testing demonstrated suppressed TSH (0.171 µIU/mL) with markedly elevated thyroid hormone levels, including free T4 >4.0 ng/dL (assay upper limit) and free T3 >18 pg/mL (assay upper limit), raising concern for severe exogenous thyrotoxicosis with impending thyroid storm.

Physical examination

Representative examination during endocrine evaluation showed a confused but arousable patient in no acute respiratory distress. Cardiovascular exam demonstrated tachycardia with normal S1/S2. Lungs were clear to auscultation. The abdomen was soft and non-tender. Extremities showed no edema. Neurologic exam was notable for confusion and tremor, with spontaneous movement of all extremities. He remained afebrile.

Diagnostic workup

A portable chest radiograph showed no acute cardiopulmonary process, and a non-contrast computed tomography (CT) of the head demonstrated no acute intracranial abnormality without hemorrhage or mass effect. High-sensitivity troponin-I was <4 ng/L. Thyroid-related evaluation supported an exogenous etiology: thyroglobulin was 11.4 ng/mL, thyroid-stimulating immunoglobulin (TSI) was negative/low, and thyroglobulin antibody was negative/low.

Management

Early management included corticosteroids and brief antithyroid therapy (propylthiouracil (PTU) and methimazole were administered before endocrine reassessment), though antithyroid drugs were not continued after the recognition of an exogenous hormone source. The patient was admitted to a monitored setting with telemetry. Beta-blockade with metoprolol 25 mg twice daily was used for rate control and blood pressure management.

Given persistent clinical instability with severe biochemical thyrotoxicosis and the anticipated prolonged intravascular burden of protein-bound levothyroxine, TPE was pursued as a hormone-removal strategy. Access was achieved via fluoroscopic placement of a 13-French, 15 cm non-tunneled trialysis catheter into the right internal jugular vein, with the catheter tip confirmed in the inferior superior vena cava, and the line was used immediately for the initiation of TPE. The patient received five sessions of TPE on hospital days 3-7, with serial monitoring of thyroid indices following sessions. Adjunctive therapy included dexamethasone 4 mg every 12 hours to inhibit the peripheral conversion of T4 to T3 and cholestyramine 4 g twice daily to interrupt the enterohepatic recycling of thyroid hormone. Both were used transiently and discontinued as the patient stabilized. Episodes of agitation were managed with haloperidol as needed, and psychiatry maintained safety precautions, including one-to-one observation.

Clinical and biochemical response

Serial thyroid function indices by hospital day, aligned to TPE sessions, are summarized in Table 2.

**Table 2:** Thyroid function indices by hospital day relative to TPE sessions Reference ranges: FT4: 0.70-1.48 ng/dL; FT3: 1.58-3.91 pg/mL; TSH: 0.35-4.94 µIU/mL. Values reported as exceeding assay upper limits are shown as ">4.00" (FT4) and ">18.00" (FT3). TPE sessions occurred on hospital days 3-7. TPE: therapeutic plasma exchange; FT4: free thyroxine; FT3: free triiodothyronine; TSH: thyroid-stimulating hormone; Y: yes; N: no

Hospital day	TPE performed?	FT4 (ng/dL)	FT3 (pg/mL)	TSH (µIU/mL)	Total T4 (µg/dL)
1	N	>4.00	-	0.171	-
2	N	-	-	-	-
3	Y	-	>18.00	-	>48
4	Y	>4.00	>18.00	-	-
5	Y	>4.00	17.87	-	-
6	Y	>4.00	11.19	-	-
7	Y	3.55	7.21	-	-
8	N	2.62	4.98	-	-
9	N	2.08	4.43	-	-
10	N	1.87	-	-	~20

These biochemical improvements paralleled clinical stabilization, with improved mental status and better control of tachyarrhythmia. At the cardiology evaluation, the patient converted to a normal sinus rhythm. Given a CHA₂DS₂-VASc score of 2, long-term anticoagulation was considered but deferred due to acute safety concerns and self-harm risk, with planned outpatient reassessment.

By the completion of five TPE sessions, the patient's hemodynamics and mental status had improved sufficiently for medical clearance planning, with the intent to transfer to inpatient psychiatry once a bed was available. Dexamethasone and cholestyramine were discontinued on transfer. The discharge plan included repeating thyroid function tests in 7-10 days and restarting physiologic levothyroxine approximately 1-2 weeks after stabilization, guided by interval thyroid function testing. A hospital day timeline summarizing key events, rhythm changes, and therapies is provided in Table 3.

**Table 3:** Hospital day timeline of key events, cardiovascular course, and therapies TPE was performed on hospital days 3-7 (five sessions total). Medication doses: metoprolol 25 mg twice daily; dexamethasone 4 mg every 12 hours; cholestyramine 4 g twice daily; haloperidol 2 mg IV every eight hours PRN for agitation. Anticoagulation was deferred due to safety risk. TPE: therapeutic plasma exchange; ED: emergency department; AF: atrial fibrillation; RVR: rapid ventricular response; RIJ: right internal jugular; IV: intravenous; PRN: as needed

Hospital day	Clinical status/key events	Rhythm/hemodynamics	Therapies/procedures	Disposition/notes
Day 1	Ingestion (~8,000 mcg levothyroxine + co-ingestants); ED presentation	Initially stable	Metabolic acidosis corrected; electrolyte replacement; psych evaluation	Admitted to a monitored setting
Day 2	Clinical deterioration: agitation, confusion developing	Tachycardia 140-150 bpm; onset of AF with RVR	Beta-blockade initiated	Telemetry/monitored unit
Day 3	Endocrine consult for thyroid storm; mental status: confused	AF with RVR persists	TPE catheter placement (RIJ); TPE session #1	No fever observed
Days 4-7	TPE sessions ongoing; clinical improvement noted	Rate control improving	TPE sessions #2-#5 (one daily); dexamethasone + cholestyramine ongoing	PRN haloperidol for agitation
Days 7-10	Clinical stabilization; mental status clearing	Conversion to sinus rhythm (noted at cardiology evaluation)	Anticoagulation deferred (safety risk)	Preparation for transfer
Transfer/discharge	Medically stable for psychiatry	Stable sinus rhythm	Dexamethasone and cholestyramine stopped	Medically cleared; transfer planning to inpatient psychiatry

**Figure 1:**
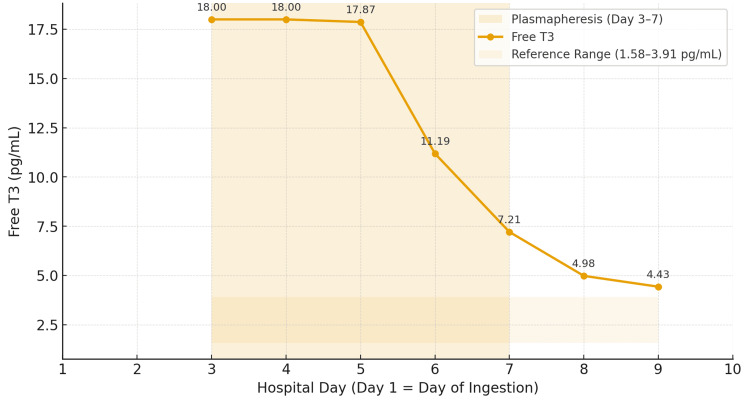
Free T3 by hospital day with plasmapheresis window Free T3 (pg/mL) by hospital day, annotated with TPE sessions (hospital days 3-7) and reference range. T3: triiodothyronine; TPE: therapeutic plasma exchange

**Figure 2:**
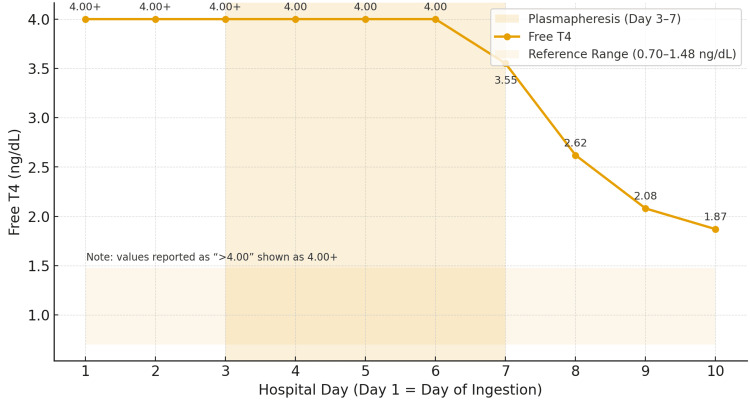
Free T4 by hospital day with plasmapheresis window Free T4 (ng/dL) by hospital day, annotated with TPE sessions (hospital days 3-7) and reference range. Values reported as ">4.00" were plotted at 4.00 to reflect assay upper limit. T4: thyroxine: TPE: therapeutic plasma exchange

**Figure 3:**
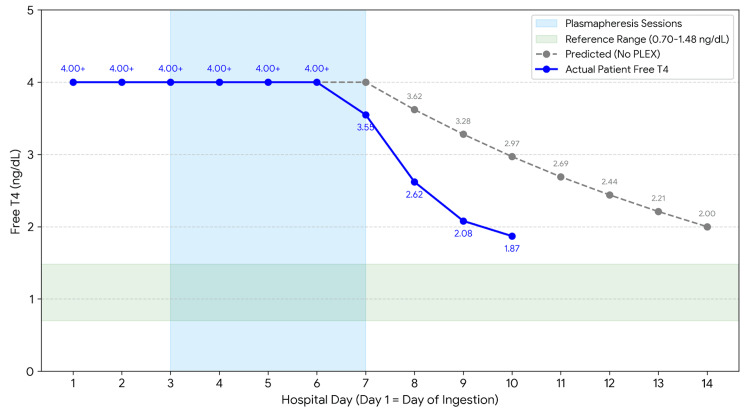
T4 clearance: patient course vs. predicted natural decay Free T4 trajectory comparing observed decline with predicted natural decay, demonstrating accelerated biochemical improvement coinciding with TPE. Predicted decay assumes first-order elimination based on levothyroxine half-life and does not account for enterohepatic recirculation, variable absorption, or ongoing conversion to T3. TPE: therapeutic plasma exchange; T3: triiodothyronine; T4: thyroxine

Trends in free T3 and free T4 by hospital day, annotated with TPE sessions, are shown in Figure 1 and Figure 2, and the observed free T4 decline compared with predicted natural decay is shown in Figure 3.

## Discussion

The management of massive levothyroxine (T4) ingestion presents a unique challenge that differs significantly from endogenous thyrotoxicosis, particularly in terms of the underlying source of the hormone and the required speed of clearance [6]. In this case, the ingestion of 8,000 mcg, approximately 80 times the patient's maintenance dose, created a massive circulating reservoir of T4. Unlike endogenous hyperthyroidism (e.g., Graves' disease), where the pathology lies in glandular overproduction, exogenous overdose bypasses the hypothalamic-pituitary-thyroid (HPT) axis, making standard antithyroid medications like methimazole largely ineffective [7].

Pathophysiology and kinetics 

Understanding the pharmacokinetics of thyroid hormones is essential to justifying the use of aggressive removal strategies like TPE. Levothyroxine is highly protein-bound (>99%), primarily to thyroxine-binding globulin (TBG), transthyretin, and albumin [8]. Because of this high degree of protein binding, T4 has a relatively small volume of distribution (approximately 0.1-0.2 L/kg), consistent with a substantial circulating protein-bound hormone that can be targeted by TPE [8]. This makes it an ideal target for TPE. While hemodialysis is of limited benefit because it cannot effectively remove large, protein-bound complexes, TPE physically removes the patient's plasma and the bound T4 complexes, replacing them with exogenous protein [9]. Previous case series have demonstrated that TPE can reduce serum free T4 levels by over 50% in a single session, a rate far exceeding the natural metabolic clearance in severe overdose [4,9].

Limitations of conventional therapy 

A critical decision in this case was the discontinuation of thionamides after early administration, once an exogenous source was recognized. These agents function by inhibiting thyroid peroxidase to prevent new hormone synthesis. In exogenous ingestion, endogenous production is already physiologically suppressed via negative feedback; thus, blocking the thyroid gland provides no benefit [10].

Management in exogenous levothyroxine overdose focuses on three principal strategies: symptomatic control, inhibition of peripheral conversion, and interruption of enterohepatic circulation. Beta-blockade, such as propranolol or cardioselective agents including metoprolol, is used for adrenergic symptom control and management of tachyarrhythmias [11]. Propranolol may provide additional benefit through the inhibition of type 1 5′-deiodinase, thereby reducing the peripheral conversion of T4 to the more active T3. Corticosteroids such as dexamethasone further inhibit peripheral T4-to-T3 conversion, mitigating biologically active hormone levels [12]. Finally, because thyroid hormones undergo enterohepatic recirculation, cholestyramine plays a critical adjunctive role by binding thyroid hormone in the gastrointestinal tract, preventing reabsorption and accelerating fecal excretion [13].

The "clinical lag" and monitoring

This case highlights the "clinical lag" inherent in T4 overdoses. Because T4 acts as a prohormone, peak metabolic effects occur only after conversion to T3 and subsequent genomic transcription, often taking 24-72 hours [14]. This explains why our patient "crashed" on hospital day 2 despite initial stability. Clinicians must resist early discharge, as observation for at least 48-72 hours is imperative to capture the delayed peak of T3-mediated toxicity [15].

Study limitations 

While this case demonstrates the successful use of TPE, there are limitations to this report. As a single case observation, the generalizability of these findings is inherently limited. Furthermore, the patient's clinical presentation was confounded by the massive co-ingestion of amphetamines and other substances, which complicated the initial diagnostic picture and may have independently contributed to the observed adrenergic symptoms, such as tachycardia and agitation.

## Conclusions

Massive levothyroxine ingestion can lead to severe, delayed-onset thyrotoxicosis that is refractory to standard antithyroid medications. This case demonstrates that TPE is a highly effective rescue therapy for severe exogenous thyrotoxicosis/impending thyroid storm after massive levothyroxine ingestion, facilitating rapid hormonal clearance by removing protein-bound T4. Furthermore, this case serves as a cautionary reminder that the initial clinical presentation of a levothyroxine overdose can be misleading. Patients should be admitted to a monitored setting regardless of early stability to manage the inevitable peak in peripheral T4-to-T3 conversion. TPE should be considered early in the clinical course when massive ingestion is suspected and life-threatening symptoms, such as tachyarrhythmias or altered mental status, begin to emerge.
